# Practices related to tobacco sale, promotion and protection from tobacco smoke exposure in restaurants and bars in Kampala before implementation of the Uganda tobacco control Act 2015

**DOI:** 10.1186/s12971-017-0129-8

**Published:** 2017-05-02

**Authors:** Steven Ndugwa Kabwama, Daniel Kadobera, Sheila Ndyanabangi, Kellen Namusisi Nyamurungi, Shannon Gravely, Lindsay Robertson, David Guwatudde

**Affiliations:** 1 Makerere University, School of Public Health, Kampala, Uganda; 2 Center for Tobacco Control in Africa, Kampala, Uganda; 3 International Tobacco Control Policy Evaluation Project, Waterloo, Canada; 4 University of Otago, Dunedin, New Zealand; 5 Department of Epidemiology and Biostatistics, Makerere University School of Public Health, Kampala, Uganda

**Keywords:** Tobacco, Uganda, Tobacco control Act, Practices, Compliance, Smoke-free, TAPS

## Abstract

**Background:**

The Word Health Organization’s Framework Convention on Tobacco Control calls on parties to implement evidenced-based tobacco control policies, which includes Article 8 (protect the public from exposure to tobacco smoke), and Article 13 (tobacco advertising, promotion and sponsorship (TAPS)). In 2015, Uganda passed the Tobacco Control Act 2015 which includes a comprehensive ban on smoking in all public places and on all forms of TAPS. Prior to implementation, we sought to assess practices related to protection of the public from tobacco smoke exposure, limiting access to tobacco products and TAPS in restaurants and bars in Kampala City to inform implementation of the new law.

**Methods:**

This was a cross-sectional study that used an observational checklist to guide observations. Assessments were: whether an establishment allows for tobacco products to be smoked on premises, offer of tobacco products for sale, observation of tobacco products for sale, tobacco advertising posters, illuminated tobacco advertisements, tobacco promotional items, presence of designated smoking zones, no-smoking signs and posters, and observation of indoor smoking. Managers of establishments were also asked whether they conducted tobacco product sales promotions within establishments. Data were collected in May 2016, immediately prior to implementation of the smoke-free and TAPS laws.

**Results:**

Of the 218 establishments in the study, 17% (*n* = 37) had no-smoking signs, 50% (*n* = 108) allowed for tobacco products to be smoked on premises of which, 63% (*n* = 68) had designated smoking zones. Among the respondents in the study, 33.3% (*n* = 72) reported having tobacco products available for sale of which 73.6% (*n* = 53) had manufactured cigarettes as the available tobacco products. Eleven percent (*n* = 24) of respondents said they conducted tobacco promotion within their establishment while 7.9% (*n* = 17) had promotional items given to them by tobacco companies.

**Conclusion:**

Hospitality establishments in Kampala are not protecting the public from tobacco smoke exposure nor adequately limiting access to tobacco products. Effective dissemination of the Tobacco Control Act 2015 is important in ensuring that owners of public places are aware of their responsibility of complying with critical tobacco control laws. This would also likely increase self-enforcement among owners of hospitality establishments and public patrons of the no-smoking restrictions.

## Background

Article 8 of the World Health Organization’s Framework Convention on Tobacco Control (WHO FCTC) states that “all countries recognize that exposure to tobacco smoke causes death, disease and disability while all parties are obligated to adopt and implement effective legislative, executive, administrative or other measures to provide protection from tobacco smoke exposure in indoor work places, public transport, indoor public places and other public places” [1]. Although the convention has been signed and ratified by most UN member states, implementation at the national level remains a prerogative of the individual FCTC Parties through the development of comprehensive tobacco control laws. A review that assessed the effect of legislative bans on the reduction of exposure to tobacco smoke established that the bans reduced exposure to tobacco smoke in work places, restaurants and pubs [2]. Another review of policies in 30 developing countries also concluded that comprehensive advertising and promotion bans resulted in a 23.5% reduction in per capita tobacco consumption, but only a 13.6% reduction with more limited bans [3]. The WHO recognizes that in order for tobacco control laws to be meaningful and comprehensive, it must include the total elimination of all forms of TAPS. Therefore, Parties to the WHO FCTC are required to adopt and implement a comprehensive ban on TAPS within 5 years after entry into force of the WHO FCTC [4]. However because the WHO FCTC has to be domesticated to the local context and because of the ever present threat of the tobacco industry undermining, diluting, and circumventing policy formulation [5], some countries like the Netherlands developed policies that were largely opposed by the public [6] while others like China have completely failed to meet their WHO FCTC obligations [7].

On 19 September 2015 the President of Uganda signed the Tobacco Control Act 2015 into law [8]. The goal of the act is to mitigate the negative public health consequences of tobacco use and exposure to tobacco smoke. The law mandates that all indoor public places, work places and public transport should be 100% smoke free and that smoking should be done 50 m away from every public place. The Act also bans all forms of tobacco advertising, sponsorship and promotion and obligates public places to institute visible no-smoking signs and posters.

The most recent Uganda Global Adult Tobacco Survey 2013 showed that 1 in every 5 adults who work indoors had experienced exposure to tobacco smoke [9] while another survey in Kampala showed that 12.1% of current smokers usually smoked in public places, 10.4% usually smoked at social events [10]. The Uganda government had previously banned tobacco advertising and promotion on state media in 1995, however in 2002, data from the Global Youth Tobacco Survey showed that almost 60% of people in that survey had seen a tobacco promotion advertisement on a billboard [11]. The current tobacco control legislation in Uganda includes The Tobacco (Control and Marketing) Act 1967 and the National Environment (control of smoking in public places) Regulations 2004. The Tobacco Control And Marketing Act regulates the production and marketing of the tobacco leaf while the National Environment control of smoking in public places regulations of 2004 prohibit the use of tobacco in an enclosed public place and stipulate that the owner of a public place should designate an area where smoking can take place. Currently, the extent to which owners of hospitality establishments institute measures to protect the public from tobacco smoke is not known considering that 62.3% of adults have experienced tobacco smoke exposure in hospitality venues in Uganda [9]. This study was aimed at describing practices related to protection of the public from tobacco smoke exposure as well as limiting access to tobacco products and tobacco advertising, promotion and sponsorship in restaurants and bars in Kampala Uganda prior to the implementation of Uganda’s Tobacco Control Act 2015.

## Methods

### Study design

The study utilized a cross sectional study design. The observations took place at restaurants and bars within Kampala District, the capital of Uganda.

A restaurant was described as any public place where food and alcohol are sold and consumed, including an area, permanent or temporary, fixed or mobile, that is accessible to the general public. A bar carried the same definition except a bar sold alcohol but not food. Shops or any other places that were primarily retail establishments albeit places where food and alcohol are sold and consumed were excluded from the study. The idea was to include only places primarily hospitality establishments.

### Sample size calculation

Kampala is administratively divided into 99 parishes [12]. For the purposes of this study, a parish was considered a cluster. A parish is the smaller administrative unit after the district and division and before the village. We therefore used the formula by Bennet et al [13] for calculating the cluster size and final sample size when the population is divided into clusters [14]. We calculated the cluster size C = P(1-P)D/S^2^B; where C is the cluster size or number of clusters; P is 0.49 - the expected proportion of bars and restaurants in breach of the existing smoke-free legislation [15], S is a standard error of 0.05; B = 6 - the number of establishments that can be visited in a cluster basing on practical grounds [13] and D is the design effect = 2 as has been used in cluster random survey sampling elsewhere [16]. The use of this design effect was to account for the 2 stage sampling procedure that reduces the precision when compared with simple random sampling. We also inflated the calculated cluster size after anticipating a refusal rate of 5%.

### Sampling procedure

At the parish, the research assistant identified the place with the highest concentration of restaurants and bars from which they selected a minimum of 6 establishments. This method has been used elsewhere because it promises greater impact [14] compared to when the establishments are chosen randomly. The rationale for this is that restaurants and bars are not randomly distributed within the parish. Some places are more residential while others will be more likely to have the restaurants and bars.

### Data collection

Data were collected in the month of May 2016. The study utilized a checklist to guide observations. Relevant questions were adapted from published guides of conducting studies related to tobacco legislation [17, 18] and also translated to Luganda. The most commonly spoken language around Kampala is Luganda and research assistants had to have the ability to communicate effectively in both English and Luganda. The interviews were carried out in either English or Luganda, depending on the choice of the respondent. The questionnaire was then entered into an electronic data collection tablet.

Prior to data collection, research assistants attended a 1-day training workshop where they were introduced to the study questionnaire and electronic data collection. The research assistants also pre-tested the questionnaire to check for correctness and clarity of questions before going to the field to collect data.

At the hospitality establishment, the research assistants made observations then filled in a checklist. Depending on the size of the establishment, observations lasted between 5 and 10 minutes. Thereafter, they asked managers about tobacco advertising, sponsorship and promotion within the hospitality establishment.

Current practices related to protection of the public from tobacco smoke exposure included whether the establishment allows for tobacco products to be smoked on the premises, presence of a designated smoking zone, reaction of the manager in charge when someone smokes in places they are not supposed to, whether the establishment has no-smoking signs and posters, and whether no-smoking signs and posters are visible at a distance of 3 m. Research assistants also looked out for observation of in-door smoking and smell of tobacco smoke on premises. The practices related to limiting access to tobacco products included whether the establishment offers tobacco products for sale and observation of tobacco products for sale. Practices related to tobacco promotion, advertising and sponsorship were assessed by observing whether tobacco advertising posters are available, whether illuminated tobacco advertisements are available and whether any tobacco promotion items are available.

### Statistical analysis

Data were exported from the data collection tablet into Microsoft Excel for cleaning. The data were later exported from Microsoft Excel to Epi Info V7 for analysis. Descriptive analyses were done to summarize all variables by calculating the percentage of each variable. We used the Pearson’s Chi-square statistic to assess for differences between groups.

## Results

### Characteristics of the establishments

The cluster size calculation yielded a total of 35 clusters from which a minimum of 6 establishments were chosen per cluster. As such, the study involved observations from 218 establishments around Kampala City. The highest number of establishments was from Makindye Division 26.9% (58) and the lowest from Lubaga Division 15.3% (33) (Table 1).

**Table 1:** Characteristics of establishments involved in the study

Characteristic	-n-(%)
Division	
Makindye	58 (26.9)
Kawempe	42 (19.4)
Central	41 (19.0)
Lubaga	33 (15.3)
Type of establishment	
Bar or pub	140 (64.8)
Restaurant and bar	72 (33.3)
Restaurant	4 (1.9)
Nature of establishment	
Permanent structure	185 (85.7)
Semi-permanent structure	22 (10.2)
Temporary/Make-shift structure	9 (4.2)
Structure of establishment	
Both indoor and outdoor facilities	142 (65.7)
Only enclosed/indoor facilities	59 (27.3)
Only outdoor facilities	10 (4.6)
Make-shift structure	5 (2.3)
Restricts entrance for minors	119 (55.1)
Offers alcohol for sale	211 (97.7)
Offers food for sale	125 (57.9)

When we assessed for differences in the groups of establishments that sold alcohol and food, we found that 121 (56%) sold both alcohol and food, while 90 (41.7%) sold alcohol but not food. The Pearson’s Chi-square statistic did not show a statistically significant difference between establishments that sold alcohol and those that did not (*p* = 0.311).

### Practices related to protection from tobacco smoke exposure

Half of the establishments 50% (*n* = 108) allowed for tobacco products to be smoked on the premises and of these, 63% (*n* = 68) had designated smoking zones. Among those that had outdoor facilities, smoking was allowed in any outdoor area of 13.8% (*n* = 21) of these (Table 2).

**Table 2:** Assessment of practices related to protection of the public from tobacco smoke exposure

Practice	-n- (%)
Allow for tobacco products to be smoked on premises	108 (50.0)
Has a designated smoking zone	68 (63.0)
No-smoking signs and/or posters are visible	37 (17.0)
No-smoking signs and/or posters are visible within 3 m	31 (14.2)
Indoor smoking policy on premises	
Smoking is allowed anywhere	19 (8.8)
Smoking allowed in some indoor areas	27 (12.5)
Smoking not allowed in any indoor areas	168 (77.8)
Declined to answer	2 (1.0)
Outdoor smoking policy on premises	
Smoking is allowed anywhere	21 (13.8)
Smoking is allowed in some outdoor areas	78 (51.3)
Smoking not allowed any outdoor areas	52 (34.2)
Declined to answer	1 (1.0)
Action in case someone smokes where they are not supposed to	
Ask person to go to designated smoking zone	85 (39.4)
Ask person to stop smoking	54 (25.0)
Ask person to leave premises	97 (44.9)
Do nothing	23 (10.7)
Declined to answer	1 (1.0)

### Practices related to sale of tobacco products

Among the respondents in the study, 33.3% (*n* = 72) mentioned that they had tobacco products available for sale of which 73.6% (*n* = 53) had manufactured cigarettes as the available tobacco products. None of the establishments had electronic-cigarettes or smokeless tobacco products (Table 3).

**Table 3:** Current practices related to tobacco product availability and point-of-sale advertising

Practice	-n- (%)
Tobacco products are available for sale	72 (33.3)
Tobacco products available for sale	
Manufactured cigarettes	53 (73.6)
Shisha	43 (59.7)
E-cigarettes	0 (0)
Smokeless tobacco products	0 (0)
Other	2 (2.8)
Tobacco products are visible for sale	54 (24.8)
Tobacco products are displayed on organized shelf or wall	27 (12.4)

### Practices related to tobacco advertising, promotion and sponsorship

In the assessment of current practices related to tobacco advertising, promotion and sponsorship, 11% (*n* = 24) of respondents mentioned that they conduct tobacco promotion within their establishment while 7.9% (*n* = 17) had promotion items given to them by tobacco companies (Table 4).

**Table 4:** Practices related to tobacco advertising, promotion and sponsorship in the establishments

Practice	-n- (%)
Conducts tobacco product promotion within establishment	24 (11.0)
Has tobacco promotion items given bytobacco companies	17 (7.9)
Tobacco promotion and sponsorship signs and products are visible	24 (11.0)
Backlit or illuminated tobacco advertisements are visible	8 (3.7)

## Discussion

The study found that half of the establishments allowed for tobacco products to be smoked on the premises of which 63% had designated smoking zones. Although there are efforts by owners of public places to protect the public from tobacco smoke exposure, the public needs to be educated about the ineffectiveness of designated smoking zones in achieving this objective. The guidelines for implementing Article 8 of the WHO FCTC assert that there is no safe level of second-hand smoke exposure and any engineering approaches such as ventilation, air exchange or designated smoking zones are ineffective in protecting against exposure to tobacco smoke [1]. A study that compared the concentrations of respirable particles and nicotine in no-smoking and smoking sections of restaurants found that although the differences in concentrations were significant, there was incomplete protection from tobacco smoke exposure in the no-smoking sections [19]. Although the existing legislation was the National Environment control of smoking in public places regulations of 2004 that prohibits the use of tobacco in an enclosed public place and stipulates that the owner of a public place should designate an area where smoking can take place; the Tobacco Control Act 2015 completely bans smoking in any place that is accessible to the public [8], something owners of public places need to be made aware of. The law can be disseminated through forging partnerships with stakeholders such as civil society organizations and media outlets that will package the law in a form that can be consumed by the public.

The study also found that although 50% of establishments did not allow for products to be smoked on the premises, only 17% had noticeable no-smoking signs. No-smoking signs and posters are an important deterrent to initiation of smoking in an area where it is prohibited because they convey the rule that smoking is not allowed without necessarily conveying the fact that there is a law against it [20]. Although some studies have found minimal changes in smoke exposure after placement of no-smoking signs [21, 22] and worse still when there is minimal enforcement [22], others have found evidence of less smoking when the signs are instituted [23] and penalties for violations clearly stated. As the new legislation is being disseminated, owners of public places need to be informed of their responsibility to place no-smoking signs as a deterrent for people from smoking on their premises. This should be fortified with strict enforcement to ensure compliance.

Among the establishments that had tobacco products available for sale, 73.6% had manufactured cigarettes while 59.7% had ‘Shisha’ – a form of tobacco use in which flavored tobacco is inhaled through a water pipe. As most of the tobacco being accessed is manufactured tobacco, increasing taxes on tobacco products might be an effective way of limiting its access to and use by the public. Tax increases have been shown to have a direct influence on the level of access to and use of tobacco products particularly among the youth [24]. The high level of availability of flavored tobacco inhaled through a water pipe or ‘Shisha’ is noteworthy. Its’ perilous nature is such that it involves inhalation of a high amount of carbon monoxide produced when charcoal is used to heat the mixture of tobacco and molasses [25]. Shisha use has in fact been associated with carbon monoxide poisoning [26], pulmonary disease, coronary artery disease, and pregnancy related complications [27]. Enforcement will prove difficult, considering the complex nature of understanding the harms associated with Shisha and yet the new law completely bans the import, manufacture or sale of a water pipe tobacco delivery system (shisha), including the water pipe device or the water pipe tobacco product or other substances to be used in that system [8]. Law enforcers should be trained to understand and appreciate the harm due to the use of Shisha by the public.

In the study, 11% of respondents said they conducted tobacco product promotion within their establishment while tobacco advertising, promotion and sponsorship signs and products were visible in 11% of the establishments assessed. Tobacco product marketing through advertising, promotion and sponsorship promotes the initiation, continuation and re-uptake of smoking because it fosters positive attitudes, beliefs and expectations regarding tobacco use [28]. The WHO has called on countries that are party to the FCTC to institute legislation that completely bans any form of tobacco advertising, promotion or sponsorship [1]. It has been shown that regulation of tobacco product marketing significantly decreases smokers’ awareness of pro-smoking cues [29]. Pursuant to recommendations by the WHO FCTC, Uganda’s Tobacco Control Act provides for a comprehensive ban on all forms of tobacco advertising, promotion and sponsorship. The public needs to be informed about subtle forms of tobacco marketing such as offers of non-tobacco products that resemble tobacco products, provision of financial and other support to owners of hospitality establishments, product diversification through brand stretching and brand sharing, brand marking in hospitality establishments and a lot more that can be employed by the tobacco industry.

New legislations are only as good as the effectiveness with which they are enforced. However, the uniqueness of the Tobacco Control Act is that if managers and owners of hospitality establishments understand the perils of exposure to tobacco smoke, implementation of the law can rely on self-enforcement. This study revealed that only 25% of managers of establishments would ask someone to stop smoking if they smoked where they were not supposed to and 11% would do nothing. Self-enforcement involves managers of hospitality establishments either stopping anyone smoking in their premises or asking them to leave the premises. It is important that the dissemination of the new law to owners of public places covers issues to do with the dangers of inhaling tobacco smoke and the risks of disease and disability from exposure to tobacco smoke. The government could also partner with non-governmental organizations involved in tobacco control as well as associations such as hospitality industry associations that could provide effective platforms for disseminating the law. This would increase the possibility of self-enforcement in hospitality establishments.

### Limitations

The findings of the current study are subject to a number of limitations. The study was purely quantitative and based on an observational checklist and lacks a deeper understanding of views of various stakeholders. However, the main objective of the study was to assess practices and provide baseline data which will be used to assess change in behavior regarding protecting the public from tobacco smoke. In addition, the findings of the study are only generalizable to the population of Kampala. However, although findings are generalizable to one district, the policy implications and how the data can inform implementation can be applied to other districts in Uganda.

## Conclusions

The study has revealed that prior to implementation of the Tobacco Control Act, the practices in hospitality venues do not suffice to protect the public from tobacco smoke exposure. Rather the current practices promote tobacco use through point of sale advertising.. When not prohibited, it is common practice that the tobacco industry carries out TAPS activities in hospitality establishments. With the coming into force of the Uganda Tobacco Control Act 2015, effective dissemination of the law to hospitality venues and the public will be key in ensuring compliance and more so demand for clean environments by the public. Findings from the study also provide a baseline for future assessment of the failure or success of the legislation in terms of reducing exposure to tobacco smoke.

## Abbreviations

WHO: World Health Organization
GATS: Global adult tobacco survey
FCTC: Framework convention on tobacco control
